# Supplementary material for: Differential expression of tissue-restricted antigens among mTEC is associated with distinct autoreactive T cell fates
Source: Nat Commun. 2020 Jul 24;11:3734. doi: 10.1038/s41467-020-17544-3 (PMC7381629; doi:10.1038/s41467-020-17544-3)
Supplement: Supplementary file 1 — Supplementary Information [file 41467_2020_17544_MOESM1_ESM.pdf]

Supplementary information

Differential expression of tissue-restricted antigens among mTEC is associated with distinct autoreactive T cell fates

Lebel *et al*.

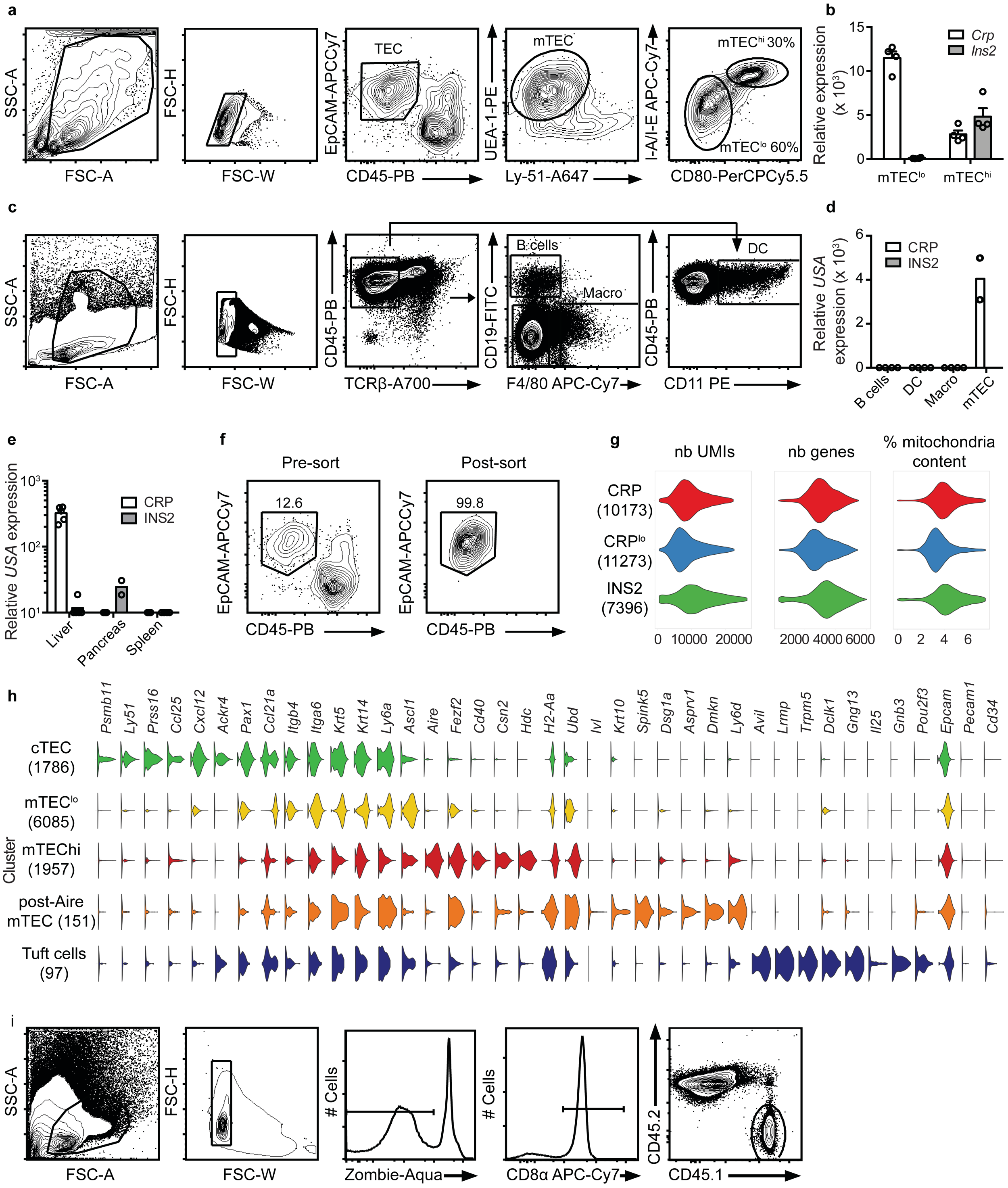

Supplementary Fig. 1. Differential expression of tissue-restricted antigens among mTEC subsets. (**a**) Gating strategy for sorting thymic epithelial cells (TEC) (EpCAM$^+$CD45$^-$), total medullary thymic epithelial cells (mTEC) (EpCAM$^+$CD45$^-$UEA-1$^+$Ly51$^-$), mTEC^{hi} (EpCAM$^+$CD45$^-$UEA-1$^+$Ly51$^-$I-A/I-E^{hi}CD80^{hi}), and mTEC^{lo} (EpCAM$^+$CD45$^-$UEA-1$^+$Ly51$^-$I-A/I-E^{lo}CD80^{lo}) cells for qPCR analysis presented in Fig. 1b-d and Supplementary Fig.1b, single-cell RNA sequencing data presented in Fig. 1e-i and Supplementary Fig. 1g,h and *in vitro* culture experiment in Supplementary Fig. 3e,f. (**b**) Relative expression of *Crp* and *Ins2* in sorted mTEC^{lo} and mTEC^{hi} cells from WT mice. The thymus from 3 mice were pooled prior to sorting (mean

+/- SEM, n = 4 experiment per group). (**c**) Gating strategy for sorting thymic B cells ($CD45^+TCR\beta^-CD19^+$), dendritic cells ($CD45^+TCR\beta^-CD11c^+$), and macrophages ($CD45^+TCR\beta^-F4/80^+$) for qPCR analysis presented in Supplementary Fig. 1d. (**d**) Relative *USA* expression in sorted thymic B cells, dendritic cells (DC), and macrophages (Macro) from CRP and INS2 Tg mice. mTEC from CRP Tg mice were included as a positive control (n = 2 mice per group). (**e**) Relative *USA* expression in the liver (n = 5 mice per group), the pancreas (n = 3 CRP and 2 INS2 mice), and the spleen (n = 3 CRP and 4 INS2 mice) from CRP and INS2 Tg mice. (**f**) Representative purity assessment before and after sort for single-cell RNA sequencing of CRP, CRP^{lo}, and INS2 Tg mice. (**g**) Distribution of quality control statistics per cell for the three transgenic mice. Number of sequenced cells are indicated in parentheses. UMIs, Unique Molecular Identifiers. Data are from one experiment with one mouse per group. (**h**) Gene expression distribution for selected cell type specific canonical markers of TEC isolated from CRP Tg mice. Number of cells are indicated in parentheses. Violins are colored by label and scaled to the same width. (**i**) Gating strategy for the analysis of the OT-I cells ($CD8^+CD45.1^+$) overlaid in the thymic slices presented on Fig. 1j-m. Source data are provided as a Source Data file.

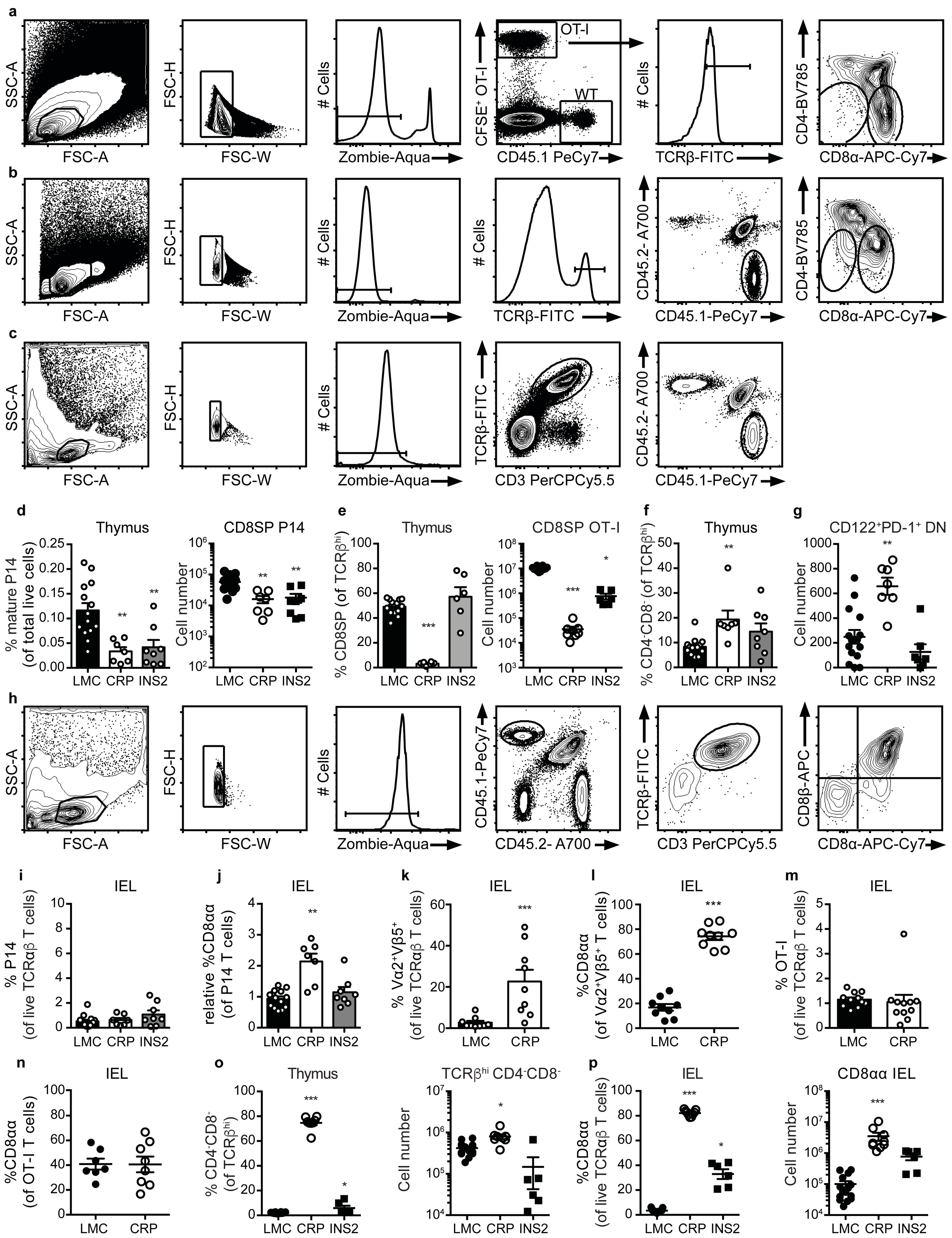
a
SSC-A
FSC-A
FSC-H
FSC-W
Cells
Zombie-Aqua
CFSE+ OT-I
CD45.1 PeCy7
OT-I
WT
TCRβ-FITC
CD4-BV785
CD8α-APC-Cy7
b
CD45.2- A700
CD45.1-PeCy7
c
TCRβ-FITC
CD3 PerCPCy5.5
d
Thymus
CD8SP P14
% mature P14 (of total live cells)
Cell number
LMC CRP INS2
e
Thymus
CD8SP OT-I
% CD8SP (of TCRβhi)
f
Thymus
% CD4-CD8- (of TCRβhi)
g
CD122+PD-1+ DN
h
CD45.1-PeCy7
CD45.2- A700
CD8β-APC
i
IEL
% P14 (of live TCRαβ T cells)
j
IEL
relative %CD8αα (of P14 T cells)
k
IEL
% Vα2+Vβ5+ (of live TCRαβ T cells)
LMC CRP
l
IEL
%CD8αα (of Vα2+Vβ5+ T cells)
m
IEL
% OT-I (of live TCRαβ T cells)
n
IEL
%CD8αα (of OT-I T cells)
o
Thymus
% CD4-CD8- (of TCRβhi)
TCRβhi CD4-CD8-
p
IEL
%CD8αα (of live TCRαβ T cells)
CD8αα IEL

Supplementary Fig. 2. Developmental fate of MHC class I-restricted autoreactive T cells in CRP and INS2 Tg mice. (**a**) Gating strategy for the analysis of OT-I thymocytes ($CFSE^+CD45.1^-$) overlaid on thymic slices presented in Fig. 2a-d and Fig. 3a-d. (**b-d and f-j**) Littermate control (LMC), CRP, and INS2 recipient mice (CD45.2) were irradiated and reconstituted with 1% TCR Tg (CD45.1) and 99% WT (CD45.1.2) bone marrow. Gating strategy for the analysis of TCR Tg ($TCR\beta^+CD45.1^+CD45.2^-$) cells in the thymus (**b**) (Fig. 2e, Fig. 3e,f and l and Supplementary Fig. 2d-g and o) and the lymph nodes (**c**) (Fig. 2f). (**d**) Flow cytometry analysis of the proportion (left panel) and cell number (right panel) of mature $TCR\beta^{hi}$ $CD8^+$ SP P14 thymocytes (mean +/- SEM, n = 14 LMC, 7 CRP, and 8 INS2 mice). (**e**) CRP and INS2 Tg mice were crossed to OT-I TCR Tg mice. Flow cytometry analysis of the proportion (left panel) and number (right panel) of mature $TCR\beta^{hi}$ $CD8^+$ SP OT-I thymocytes (mean +/- SEM, n = 14 LMC, 8 CRP, and 6 INS2 mice). (**f**) Compilation of the proportion of $CD4^-CD8^-$ cells within the live $TCR\beta^{hi}$ P14 thymocyte population and (**g**) number of $CD122^+PD\text{-}1^+$ DN P14 cells. (**h**) Gating strategy for the analysis of TCR Tg ($TCR\beta^+CD45.1^+CD45.2^-$) cells in the intraepithelial lymphocytes of the small intestine (Fig. 3g-k and Supplementary Fig. 2i-n and p). Proportion of P14 cells of $TCR\alpha\beta$ intraepithelial lymphocytes (IEL) from the small intestine (**i**) and the proportion of $TCR\alpha\beta^+$ $CD8\alpha\alpha$ cells of P14 T cells (**j**) (f-j, mean +/- SEM, n = 14 LMC, 7 CRP, and 8 INS2 mice). (**k, l**) LMC and CRP recipient mice (CD45.1.2) were irradiated and reconstituted with 1% OT-I $\beta2M^{-/-}$ (CD45.2, $V\alpha2^+V\beta5^+$) and 99% $\beta2M^{-/-}$ (CD45.2) bone marrow, and the proportion of $V\alpha2^+V\beta5^+$ cells within the $TCR\alpha\beta$ IEL from the small intestine (**k**) and the proportion of $TCR\alpha\beta^+$ $CD8\alpha\alpha$ IEL of $V\alpha2^+V\beta5^+$ T cells (**l**) were analyzed 6-8 weeks post-reconstitution (mean +/- SEM, n = 9 mice per group). Mature OT-I T cells were adoptively transferred into LMC and CRP mice, and the proportion of OT-I T cells of $TCR\alpha\beta$ IEL (**m**) (mean +/- SEM, n = 11 mice per group) and the proportion of $TCR\alpha\beta^+$ $CD8\alpha\alpha$ cells of OT-I T cells (**n**) (mean +/- SEM, n = 7 LMC and 8 CRP mice) were analyzed 6-8 weeks post-transfer. CRP and INS2 Tg mice were crossed to OT-I TCR Tg mice and (**o**) the proportion (left panel) and absolute number (right panel) of $TCR\beta^{hi}CD4^-CD8^-$ DN cells (mean +/- SEM, n = 14 LMC, 8 CRP, and 6 INS2 mice) as well as (**p**) the proportion (left panel) and absolute number (right panel) of $CD8\alpha\alpha$ IEL were analyzed (mean +/- SEM, n = 15 LMC, 9 CRP, and 6 INS2 mice). Data are pooled from two to three independent experiments. Statistical analyses were performed by Kruskal-Wallis test followed by Dunn's post-hoc comparisons to LMC, two-sided (**d-g, i, j, o, p**) and two-tailed Mann-Whitney U test (**k-n**). $^*p < 0.05$, $^{**}p < 0.01$, $^{***}p < 0.001$ as compared to LMC. Source data are provided as a Source Data file.

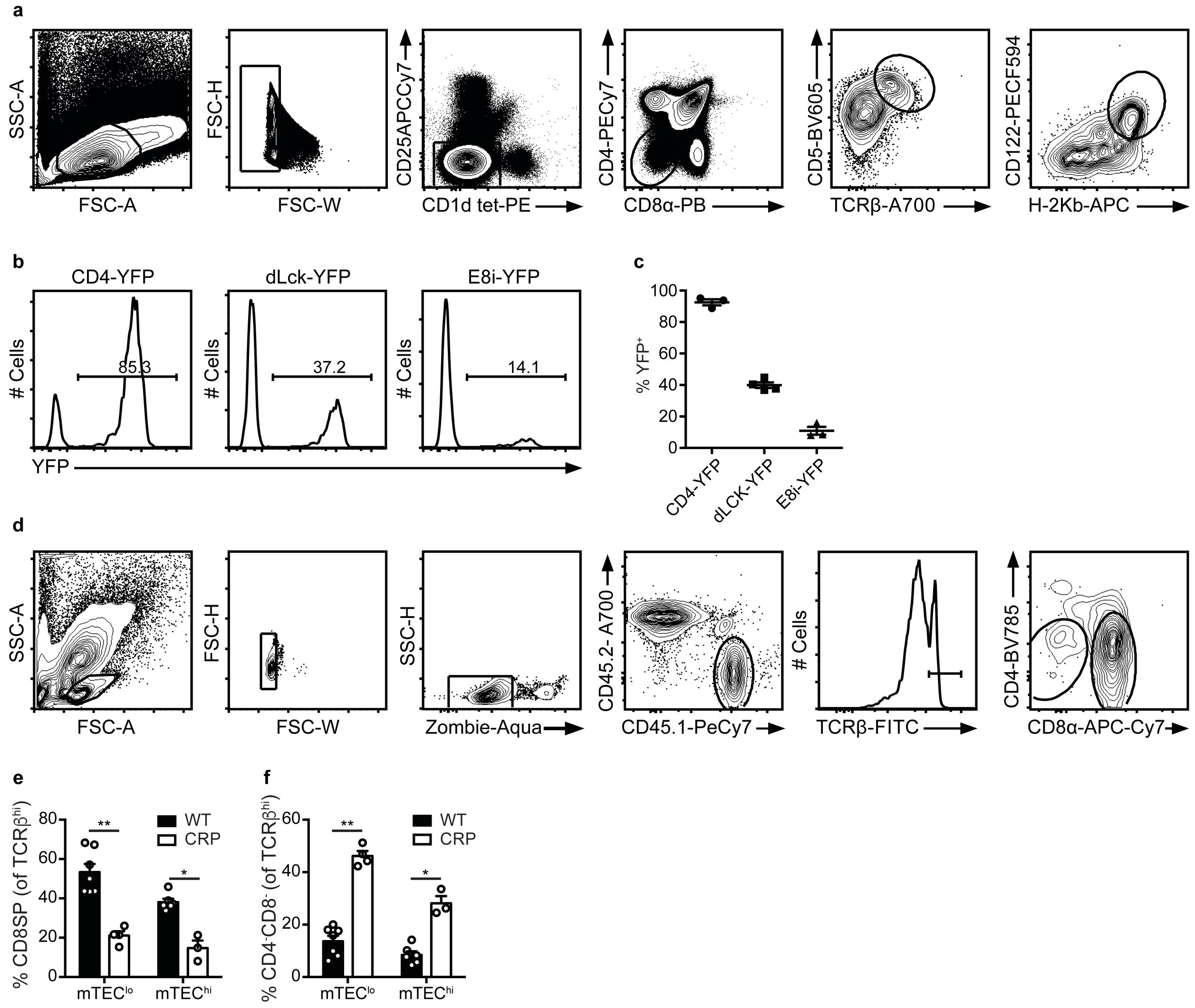

Supplementary Fig. 3. (**a**) Gating strategy for the analysis of IEL precursors (IELp, CD25$^-$CD1d tet$^-$CD4$^-$ CD8α$^-$CD5$^+$TCRβ$^+$CD122$^+$H2Kb$^+$) in the thymus for data presented in Supplementary Fig. 3b,c. (**b**) Representative histograms of YFP expression in CD4-YFP (left panel), dLck-YFP (middle panel), and E8i-YFP (right panel) mice after gating on IELp. (**c**) Compilation of the proportion of YFP$^+$ cells within IELp from CD4-YFP, dLck-YFP, and E8i-YFP mice (mean +/- SEM, n = 3 CD4-YFP, 4 dLck-YFP, and 3 E8i-YFP mice). (**d**) Gating strategy for the analysis of the OT-I thymocytes (CD45.1$^+$CD45.2$^-$) cultured with mTEC^{lo} or mTEC^{hi} cells presented in Supplementary Fig. 3e,f. (**e**) Proportion of CD8SP and (**f**) CD4$^-$CD8$^-$ DN among the TCRβ^{hi} OT-I thymocytes cultured with mTEC^{lo} or mTEC^{hi}. Data from panels **e** and **f** are pooled from at least three independent experiments and for each experiment, eight mice were pooled together to sort the mTEC$^{lo/hi}$ cells (mean +/- SEM, n = 7 mTEC^{lo} WT, 4 mTEC^{lo} CRP, 6 mTEC^{hi} WT, and 3 mTEC^{hi} CRP conditions, examined over three independent experiments). Statistical analyses were performed by two-tailed Mann-Whitney U test. *p < 0.05, **p < 0.01. Source data are provided as a Source Data file.

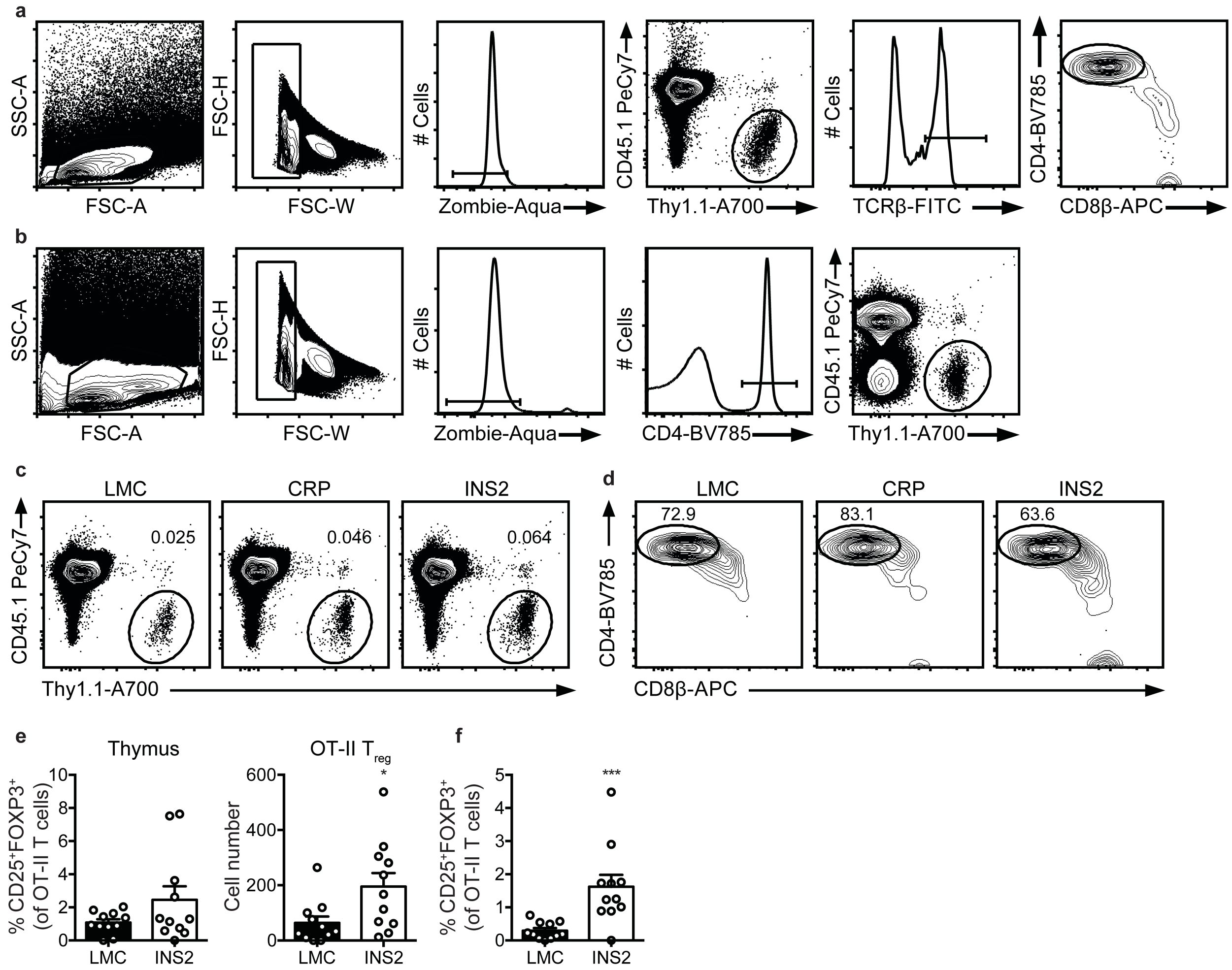

Supplementary Fig. 4. mTEC^{hi}-restricted expression of *USA* preferentially induces T_{reg} differentiation in a hematopoietic cell-independent manner. Littermate control (LMC), CRP, and INS2 mice (CD45.2, Thy1.2) were irradiated and reconstituted with 1% OT-II (CD45.2, Thy1.1) and 99% WT (CD45.1, Thy1.2) bone marrow. The hematopoietic chimeras were analyzed 6-8 weeks post-irradiation. Gating strategy for the analysis of OT-II cells (CD45.1^{-}Thy1.1^{+}) isolated from the thymus (**a**) or the lymph nodes (**b**) of the hematopoietic chimeras presented in Fig. 4a-e and Supplementary Fig. 4c-f. (**c**) Representative flow plots of CD45.1 and Thy1.1 expression on total live cells from the thymus. (**d**) Representative flow plots of CD4 and CD8β expression on live TCRβ^{hi} OT-II thymocytes. LMC and INS2 recipient mice (CD45.1.2, Thy1.2) were irradiated and reconstituted with 1% OT-II (CD45.2, Thy1.1) and 99% MHCII KO CD1d-deficient (CD45.2, Thy1.2) bone marrow. The proportion (left panel) and number (right panel) of CD25^{+} FoxP3^{+} cells of OT-II T cells in the thymus (**e**) and lymph nodes (**f**) 6-8 weeks post-irradiation (mean +/- SEM, n = 11 mice per group, examined over three independent experiments). Statistical analyses were performed by two-tailed Mann-Whitney U test. *p < 0.05, ***p < 0.001. Source data are provided as a Source Data file.

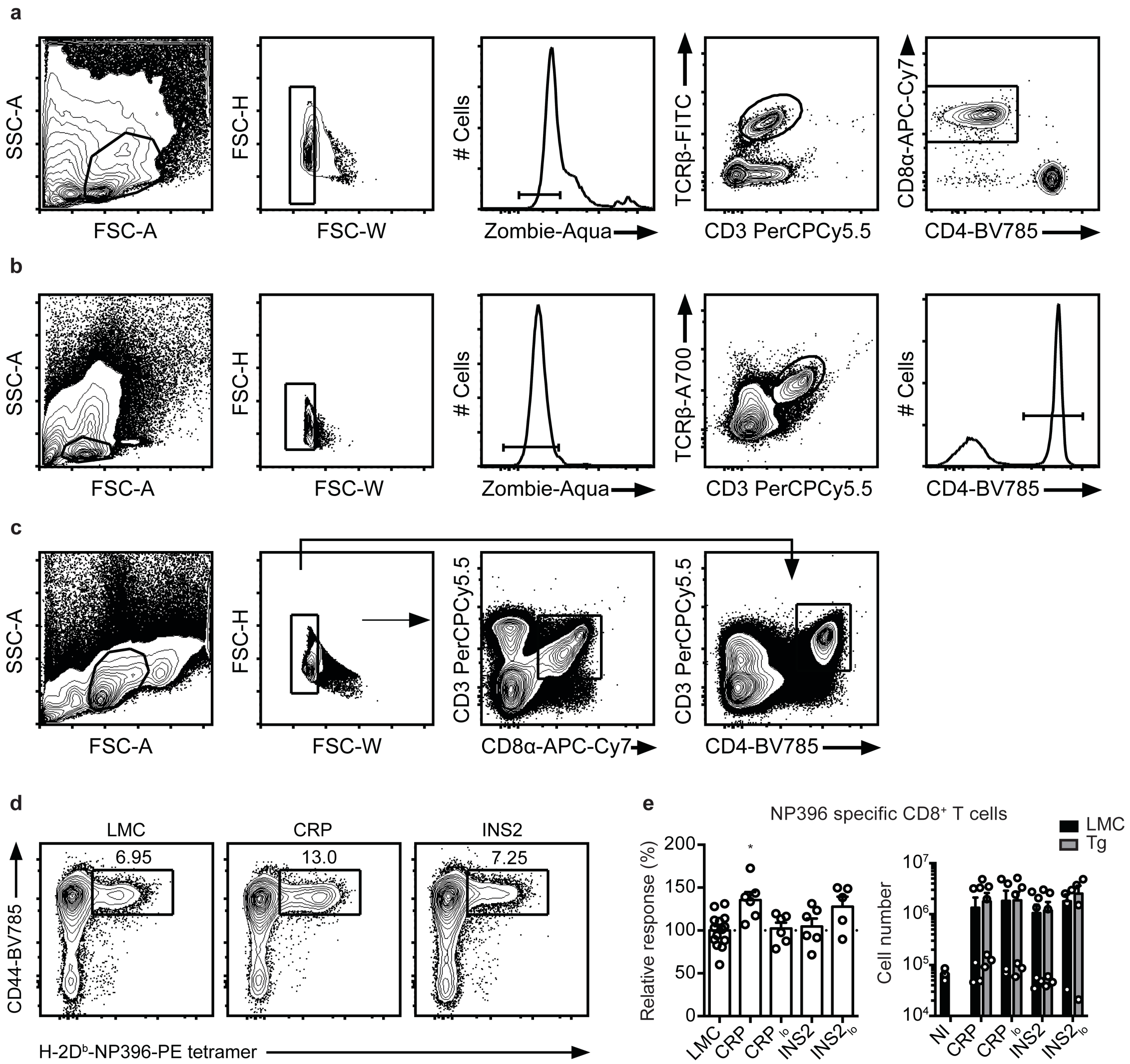

Supplementary Fig. 5. Gating strategy for the analysis of the polyclonal CD8 (**a**) and CD4 (**b**) T cell repertoire in naïve mice presented in Fig. 5a-i. (**c-e**) Mice were infected with LCMV-OVA and the antigen-specific response was measured in the spleen 8 days post-infection. (**c**) Gating strategy for the analysis of the CD4 and CD8 T cells response after LCMV infection before gating on the tetramer positive cells (corresponding to Fig. 6a-k and Supplementary Fig. 5d,e) (**d**) Representative CD44 and H-2D^b-NP396 tetramer staining on CD8$^+$ T cells from the spleens of littermate controls (LMC), CRP, and INS2 mice. (**e**) Relative response (left panel) and absolute number (right panel) of NP396 specific CD8$^+$ T cell response in *USA* expressing mice as compared to LMC (mean +/- SEM, n = 14 LMC, 6 CRP, 6 CRP^{lo}, 6 INS2, and 5 INS2^{lo} mice, examined over three independent experiments). The p values indicated are calculated by Kruskal-Wallis test followed by Dunn's post-hoc comparisons to LMC, two-sided. *$p < 0.05$ as compared to LMC. Source data are provided as a Source Data file.

		CRP	CRP^{lo}	INS
Cell counts	Number of cells before filtering	13690	13575	12583
	Number of cells after filtering	10173	11273	7396
	Proportion of cells kept with filtering	0,743097151	0,830423573	0,58777716
Thresholds	Minimal gene number per cell	252	351	250
	Maximal gene number per cell	5467	5404	5996
	Minimal mitochondrial content	0	0	0
	Maximal mitochondrial content	7,5	7,5	7,5
	Minimal UMI number per cell	0	0	0
	Maximal UMI number per cell	22358	22597	26427
QC metrics before filtering	Minimal number of genes	70	64	113
	Minimal number of UMIs	504	500	500
	Minimal mitochondrial content	0	0	0
	Maximal number of genes	8172	8026	8384
	Maximal number of UMIs	72387	59343	68417
	Maximal mitochondrial content	93,49	92,71	88,36
	Mean number of genes	2859,63	2877,53	2793,21
	Meanl number of UMIs	9343,77	9399,84	9828,16
	Mean mitochondrial content	8,29	6,41	11,99
	Standard deviation in the number of genes	1303,58	1263,44	1601,27
	Standard deviation in the number of UMIs	6507,13	6598,33	8299,6
	Standard deviation in the mitochondrial content	12,35	11,16	15,25
QC metrics after filtering	Minimal number of genes	308	358	317
	Minimal number of UMIs	504	505	500
	Minimal mitochondrial content	0	0	0
	Maximal number of genes	5467	5404	5985
	Maximal number of UMIs	22345	22597	26421
	Maximal mitochondrial content	7,5	7,5	7,5
	Mean number of genes	3138,85	2980,04	3410,99
	Mean number of UMIs	9999,71	9293,33	11849,63
	Mean mitochondrial content	4,15	3,63	4,36
	Standard deviation in the number of genes	873,92	916,35	1108,59
	Standard deviation in the number of UMIs	4445,44	4573,24	5871,55
	Standard deviation in the mitochondrial content	1,2	1,11	1,4

Supplementary Table 1. Quality control metrics of single cell RNA sequencing data. QC: quality control, UMI: Unique Molecular Identifier
