# Supplementary material for: Differential expression of tissue-restricted antigens among mTEC is associated with distinct autoreactive T cell fates
Source: Nat Commun. 2020 Jul 24;11:3734. doi: 10.1038/s41467-020-17544-3 (PMC7381629; doi:10.1038/s41467-020-17544-3)
Supplement: Supplementary file 3 — Reporting Summary [file 41467_2020_17544_MOESM3_ESM.pdf]

nature research

Corresponding author(s): Heather Melichar

Last updated by author(s): Jun 30, 2020

Reporting Summary

Nature Research wishes to improve the reproducibility of the work that we publish. This form provides structure for consistency and transparency in reporting. For further information on Nature Research policies, see Authors & Referees and the Editorial Policy Checklist .

Statistics

For all statistical analyses, confirm that the following items are present in the figure legend, table legend, main text, or Methods section.

n/a	Confirmed	
☐	☒	The exact sample size (n) for each experimental group/condition, given as a discrete number and unit of measurement
☐	☒	A statement on whether measurements were taken from distinct samples or whether the same sample was measured repeatedly
☐	☒	The statistical test(s) used AND whether they are one- or two-sided<br>*Only common tests should be described solely by name; describe more complex techniques in the Methods section.*
☒	☐	A description of all covariates tested
☐	☒	A description of any assumptions or corrections, such as tests of normality and adjustment for multiple comparisons
☐	☒	A full description of the statistical parameters including central tendency (e.g. means) or other basic estimates (e.g. regression coefficient) AND variation (e.g. standard deviation) or associated estimates of uncertainty (e.g. confidence intervals)
☐	☒	For null hypothesis testing, the test statistic (e.g. F, t, r) with confidence intervals, effect sizes, degrees of freedom and P value noted<br>*Give P values as exact values whenever suitable.*
☒	☐	For Bayesian analysis, information on the choice of priors and Markov chain Monte Carlo settings
☒	☐	For hierarchical and complex designs, identification of the appropriate level for tests and full reporting of outcomes
☒	☐	Estimates of effect sizes (e.g. Cohen's d, Pearson's r), indicating how they were calculated

Our web collection on statistics for biologists contains articles on many of the points above.

Software and code

Policy information about availability of computer code

Data collection	Flow cytometry : BD FACSDiva v8.0.3
Data analysis	Flow cytometry: FlowJo v9.3<br>Statistics: GraphPad Prism v6<br>Single cell RNA sequencing: Cell Ranger version 3.0.1, the Seurat package v2.3

For manuscripts utilizing custom algorithms or software that are central to the research but not yet described in published literature, software must be made available to editors/reviewers. We strongly encourage code deposition in a community repository (e.g. GitHub). See the Nature Research guidelines for submitting code & software for further information.

Data

Policy information about availability of data

All manuscripts must include a data availability statement. This statement should provide the following information, where applicable:

- Accession codes, unique identifiers, or web links for publicly available datasets
- A list of figures that have associated raw data
- A description of any restrictions on data availability

The single-cell RNA-sequencing data have been deposited in NCBI Gene Expression Omnibus (GEO) and is accessible through GEO Series accession number GSE153288 [https://www.ncbi.nlm.nih.gov/geo/query/acc.cgi?acc=GSE153288]. The source data underlying Fig. 1b-d, g-I, k-m, Fig. 2b-f, Fig. 3b, d, f, g, i-l, Fig. 4a, b, d, e, Fig. 5b, c, e, g, I, Fig. 6b, d, f, h-k, Fig. 7a-e, Supplementary Fig. 1c-e, Supplementary Fig. 2e-p, Supplementary Fig. 3c, e, f, Supplementary Fig. 4e, f, and Supplementary Fig. 5e are provided as a Source Data file. All other data supporting the findings of this study are available within the article and its supplementary information files, or are available upon request to the authors.

Field-specific reporting

Please select the one below that is the best fit for your research. If you are not sure, read the appropriate sections before making your selection.

☒ Life sciences ☐ Behavioural & social sciences ☐ Ecological, evolutionary & environmental sciences

For a reference copy of the document with all sections, see nature.com/documents/nr-reporting-summary-flat.pdf

Life sciences study design

All studies must disclose on these points even when the disclosure is negative.

Sample size	No calculations were performed to predetermine sample size. Sample size was determined to be adequate based on previous experience with similar experiments and on the magnitude and consistency of measurable differences between groups in order to reproducibly detect specific effects. Generally, two to three repeats with a minimum of 3 mice per group were used when possible. Sample size is shown in each figure legend.
Data exclusions	No data were excluded.
Replication	All attempts at replication were successful. Generally, two to three repeats with a minimum of 3 mice per group were used when possible. This information is provided in figure legends.
Randomization	Samples were not randomized. Mice were allocated to groups based on genotype. Whenever possible, animals were housed within the same cage and age-matched.
Blinding	Investigators were not blinded to mouse genotypes for planning of experiments and to ensure that appropriate sample size was achieved by sacrificing the minimum number of mice. However, for the tumor measurements, the genotype was not display to prevent bias in research.

Reporting for specific materials, systems and methods

We require information from authors about some types of materials, experimental systems and methods used in many studies. Here, indicate whether each material, system or method listed is relevant to your study. If you are not sure if a list item applies to your research, read the appropriate section before selecting a response.

Materials & experimental systems

n/a	Involved in the study	
☐	☒	Antibodies
☐	☒	Eukaryotic cell lines
☒	☐	Palaeontology
☐	☒	Animals and other organisms
☒	☐	Human research participants
☒	☐	Clinical data

Methods

n/a	Involved in the study	
☒	☐	ChIP-seq
☐	☒	Flow cytometry
☒	☐	MRI-based neuroimaging

Antibodies

Antibodies used

Anti-mouse CD3e, Biolegend, Cat# 100328, Clone# 145 2C11, lot# B218551
Anti-mouse CD4 , Biolegend, Cat# 100422, Clone# GK1.5, lot# B224943, 224943
Anti-mouse CD4 , Biolegend, Cat# 100552, Clone# RM4-5, lot# B240240, 247274
Anti-mouse CD8a, Biolegend, Cat# 100714, Clone# 53 6.7, lot# B227808, 237526
Anti-mouse CD8b, Biolegend, Cat# 126616, Clone# YTS156.7.7, lot# B219438
Anti-mouse CD25, Biolegend, Cat# 102026, Clone# PC61, lot# B195261, B220897
Anti-mouse/human CD44, Biolegend, Cat# 103059, Clone# IM7, lot# B226852, 237883
Anti-mouse CD45, Biolegend, Cat# 103125, Clone# 30-F11, lot# B223290
Anti-mouse CD45.1, Biolegend, Cat# 110730, Clone# A20, lot# B233222
Anti-mouse CD45.2, Biolegend, Cat# 109822, Clone# 104, lot# B202497, B252126
Anti-mouse CD80, Biolegend, Cat# 104722, Clone# 16-10A1, lot# B209474
Anti-mouse CD90.1, Biolegend, Cat# 202528, Clone# OX-7, lot# B224447
Anti-mouse CD16/32, Biolegend, Cat# 101321, Clone# 93, lot# B238512
Anti-mouse CD122, Biolegend, Cat# 123218, Clone# TM-b1, lot# B236204
Anti-mouse CD326, Biolegend, Cat# 118218, Clone# G8.8, lot# B222309
Anti-mouse CD279 (PD-1), Biolegend, Cat# 135231, Clone# 29F.1A12, lot# B239282, B248896
Anti-mouse I-A/I-E, Biolegend, Cat# 107630, Clone# M5/114.15.2, lot# B199458
Anti-mouse TCRb, Biolegend, Cat# 109206, Clone# H57 597, lot# B180751

Anti-mouse TCRb, Biolegend, Cat# 109224, Clone# H57 597, lot# B241002, B278726
Anti-mouse FoxP3, ThermoFisher Scientific, Cat# 53-4774-42, Clone# 150D/E4, lot# 1934576
Anti-mouse FoxP3, BDBiosciences, Cat# 562996, Clone# MF23, lot# 6019937
Anti-mouse TCR Va2, Biolegend, Cat# 127808, Clone# B20.1, lot# B179390
Anti-mouse TCR Vb5.1,5.2, Biolegend, Cat# 139508, Clone# MR9-4, lot# B231450
Anti-mouse Ly-51, Biolegend, Cat# 108312, Clone# 6C3, lot# B222434, B222435
Biotinylated Ulex Europaeus Agglutinin I, Vector Laboratories, Cat# B-1065, Clone# UEA-1, lot# Z0806
InVivoMAb anti-mouse Thy1, BioXcell, Cat# BE0212, Clone# CD90, lot# 489417S1
Anti-Mouse CD25 Purified Low Endotoxin Functional Formulation, Leinco Technologies, Cat# C1194, Clone# PC61, lot# 0518L315

Validation

All antibodies are commercially available, validated by the manufacturer, and commonly used. They were only used on species for which they have been validated by the manufacturer. All antibodies replicate the staining provided by the manufacturer.

Eukaryotic cell lines

Policy information about cell lines

Cell line source(s)

MC57G, (RRID ID : CVCL_4985, Aden DP, Knowles BB. Immunogenetics, 1976),
B16F10 (RRID ID : CVCL_0159, Fidler IJ. Cancer Res.,1975),
B16-OVA (Linardakis E, Bateman A, Phan V, et al., Cancer Res, 2002), and
L929 fibroblast cell line (Earle WR, et al., J. Natl. Cancer Inst., 1943) were kindly provided by Dr Alain Lamarre (INRS-Institut Armand-Frappier, Laval, Quebec, Canada).

Authentication

None of the cell lines used were authenticated.

Mycoplasma contamination

The cell lines were not tested for mycoplasma contamination.

Commonly misidentified lines
(See ICLAC register)

No cell lines used are listed in the database of commonly misidentified cell lines.

Animals and other organisms

Policy information about studies involving animals; ARRIVE guidelines recommended for reporting animal research

Laboratory animals

Mouse: C57BL/6, B6.SJL, Aire-/- (B6.129S2-Airetm1.1Doi/J) and b2M-/- (B6.129P2-B2mtm1Unc/J), OT-I Rag1-deficient (NIH:C57BL/6 Tg(OT I)Rag1<tm1Mom>), OT-II Rag1-deficient, P14 TCR Tg mice, MHCII-/-CD1d-/- (B6.129PS2-H2 dlabl-Ea/J x B6.129S6-Del(3Cd1d2-Cd1d1)1Sbp/J), CD4-YFP (B6.Cg-Gt(ROSA)26Sortm3(CAG-EYFP)Hze/Jv x B6.Cg-Tg(Cd4-cre)1Cwi/BfluJ), dLCK-YFP (B6.Cg-Gt(ROSA)26Sortm3(CAG-EYFP)Hze/Jv x B6.Cg-Tg(Lck-icre)3779Nik/J), E8i-YFP (B6.Cg-Gt(ROSA)26Sortm3(CAG-EYFP)Hze/Jv x C57BL/6-Tg(Cd8a-cre)1Itan/J) and CRP, CRPlo, INS2 and INS2lo transgenic mice.
Male and female mice between 4 and 12 weeks old were used unless otherwise noted.

Wild animals

No wild animals were used in the study.

Field-collected samples

The study did not involve samples collected from the field.

Ethics oversight

All animal protocols were approved by the local Animal Care Committee at the Maisonneuve-Rosemont Hospital in accordance with the Canadian Council on Animal Care guidelines.

Note that full information on the approval of the study protocol must also be provided in the manuscript.

Flow Cytometry

Plots

Confirm that:

- [x] The axis labels state the marker and fluorochrome used (e.g. CD4-FITC).
- [x] The axis scales are clearly visible. Include numbers along axes only for bottom left plot of group (a 'group' is an analysis of identical markers).
- [x] All plots are contour plots with outliers or pseudocolor plots.
- [x] A numerical value for number of cells or percentage (with statistics) is provided.

Methodology

Sample preparation

Spleens and lymph nodes were dissociated using frosted glass slides, while single cell suspensions from the thymus were prepared using a glass tissue homogenizer. Red blood cells in spleens and thymus were lysed before analysis with Ack lysis buffer (0.15 M NH4Cl, 10mM KHCO3, 0.1mM Na2EDTA). To isolate IEL from the small intestine, the connective tissue, fat, Peyer's patches and feces were removed. One cm intestine pieces were incubated in RPMI media supplemented with 3% FBS, 5mM EDTA and 0.145mg/ml DL-Dithiotreitol (Sigma-Aldrich) for 20 minutes at 37C with agitation. The IEL were then isolated by shaking the small intestine pieces in RPMI media with 2mM EDTA followed by a 30% Percoll (GE Healthcare) gradient of the

released cells. For isolation of the thymic epithelial cells, the thymus was first cut into small pieces and pipetted up and down with a wide bore 1000 ul tip until cell release was no longer detectable. The remaining tissue was digested in RPMI media supplemented with 10% FBS, 10 mM HEPES, 0.25 mg/ml papain, 0.25 mg/ml collagenase D, 0.1 mg/ml DNase for 15 min at 37C.

Instrument

Flow cytometry analyses were performed on a BDLSR Fortessa X20 or BDLSRII flow cytometer (BD Biosciences).

Software

BD FACSDiva software was used to collect the flow cytometry data. Data were then analyzed using FlowJo software (Tree Star).

Cell population abundance

40000 to 150000 cells were obtained post-sort. For the qPCR analysis, the cells were directly sorted into Trizol; purity was not assessed. For the single cell RNA sequencing, purity was approximately 99%.

Gating strategy

Fig. 1, mTEClo were gated as follows : FSC/SSC, singlets, CD45- EPCAM+, UEA-1+ Ly51-, CD80 lo I-A/I-E lo
Fig. 1, mTEChi were gated as follows : FSC/SSC, singlets, CD45- EPCAM+, UEA-1+ Ly51-, CD80 hi I-A/I-E hi
Fig. 1, TEC were gated as follows : FSC/SSC, singlets, CD45- EPCAM+
Fig. 1, CFSE-labeled OT-I T cells overlaid on top of thymic slices were gated as follows : FSC/SSC, singlets, live, CD8a+, CD45.1 +CD45.2-
Fig. 2 and 3, CFSE-labeled OT-I thymocytes overlaid on top of thymic slices were gated as follows : FSC/SSC, singlets, live, CFSE +CD45.1-
Fig. 2 and 3, OT-I thymocytes from the hematopoietic chimera were gated as follows : FSC/SSC, singlets, live, TCRb hi, CD45.1+ CD45.2-
Fig. 2 and 3, OT-I T cells from lymph nodes of the hematopoietic chimera were gated as follows : FSC/SSC, singlets, live, TCRb +CD3+, CD45.1+ CD45.2-
Fig. 3, OT-I T cells isolated from the small intestine (IEL) were gated as follows : FSC/SSC, singlets, live, CD3+TCRb+, CD45.1+ CD45.2-
Fig. 4, OT-II thymocytes from the hematopoietic chimera were identified as follows : FSC/SSC, singlets, live, TCRb hi, CD90.1+, CD45.1-
Fig. 4, OT-II T cells from the hematopoietic chimera were gated as follows : FSC/SSC, singlets, live, CD3+CD4+, CD90.1+CD45.1-
Fig. 5, cells for the tetramer analysis in naïve mice were gated as follows : FSC/SSC, singlets, live, CD3+TCRb+, and CD8+ or CD4+
Fig. 6, cells for the tetramer analysis in LCMV infected mice were gated as follows FSC/SSC, singlets, live, and CD8+ or CD4+ before visualizing the CD44+tetramer+ cells.
Supplementary Fig. 1, B cells were gated as follows : FSC/SSC, singlets, CD45+TCRb-, CD19+
Supplementary Fig. 1, Macrophages were gated as follows : FSC/SSC, singlets, CD45+TCRb-, F4/80+
Supplementary Fig. 1, Dendritic cells were gated as follows : FSC/SSC, singlets, CD45+TCRb-, CD11c+
Supplementary Fig. 3, IEL precursors were gated as follows: FSC/SSC, singlets, live, CD1d tet-CD25-, CD4-CD8a-, CD5+TCRb+, CD122+H2Kb+
Supplementary Fig. 3, OT-I thymocytes cultured with mTEC were gated as follows: FSC/SSC, singlets, live, CD45.1+CD45.2-, TCRbhi

☒ Tick this box to confirm that a figure exemplifying the gating strategy is provided in the Supplementary Information.
